# Empirical Support for ‘Hastening-Through-Re-Automatization’ by Contrasting Two Motor-Cognitive Dual Tasks

**DOI:** 10.3389/fpsyg.2018.00714

**Published:** 2018-05-25

**Authors:** Christine Langhanns, Hermann Müller

**Affiliations:** ^1^Institute of Sports Science, Department of Psychology and Sports Science, Justus Liebig University Giessen, Giessen, Germany; ^2^Nemolab, Department of Psychology and Sports Science, Justus Liebig University Giessen, Giessen, Germany

**Keywords:** hastening, automatization, rhythmic movement, motor-cognitive dual task, upper limbs, mental calculation, fNIRS

## Abstract

Motor-cognitive dual tasks have been intensely studied and it has been demonstrated that even well practiced movements like walking show signs of interference when performed concurrently with a challenging cognitive task. Typically walking speed is reduced, at least in elderly persons. In contrast to these findings, some authors report an increased movement frequency under dual-task conditions, which they call *hastening*. A tentative explanation has been proposed, assuming that the respective movements are governed by an automatic control regime. Though, under single-task conditions, these automatic processes are supervised by “higher-order” cognitive control processes. However, when a concurrent cognitive task binds all cognitive resources, the automatic process is freed from the detrimental effect of cognitive surveillance, allowing higher movement frequencies. Fast rhythmic movements (>1 Hz) should more likely be governed by such an automatic process than low frequency discrete repetitive movements. Fifteen subjects performed two repetitive movements under single and dual-task condition, that is, in combination with a mental calculation task. According to the expectations derived from the explanatory concept, we found an increased movement frequency under dual-task conditions only for the fast rhythmic movement (paddleball task) but not for the slower discrete repetitive task (pegboard task). fNIRS measurements of prefrontal cortical load confirmed the idea of an automatic processing in the paddleball task, whereas the pegboard task seems to be more controlled by processes interfering with the calculation related processing.

## Introduction

Every-day life comprises numerous situations in which we move our body while we are performing more or less challenging cognitive tasks in parallel. We are involved in a conversation while we walk, we cut vegetables while mentally calculating quantities of ingredients, we try to retrieve the remnants of the mental roadmap of the city we revisit after so many years while we drive the car, etc. Many experimental studies have looked at how both tasks interact in such situations, which will be called motor-cognitive dual task (MCDT) henceforth. These studies show that even when well-practiced movements like walking are involved, motor and cognitive tasks interfere, meaning that performance in the MCDT condition suffers compared to when each of the tasks is performed in isolation (ST: single task condition; e.g., see review by Woollacott and Shumway-Cook, 2002). Under MCDT conditions, motor tasks show reduced movement speed (walking: Holtzer et al., 2011; manual joystick tracking: Gazes et al., 2010) or higher variability (finger tapping: Wu et al., 2013; isometric manual force production: Mandrick et al., 2013a).

However, this interference is not pervasive, as for example Lindenberger et al. (2000) observed pronounced interference effects in older adults whereas younger adults did not show any sign of it in the same MCDT situation. This is interpreted as the result of a ‘posture-first’ prioritization strategy. In case of a competition for processing resources, older subjects give higher priority to the walking task in order to prevent falls (Li et al., 2001). This interpretation is supported by studies demonstrating that the prioritization is stronger if the ‘postural threat’ is increased (Lajoie et al., 1996; Brown et al., 2002; Gage et al., 2003).

However, explicit instruction to prioritize tasks may modulate this interference profile (Yogev-Seligmann et al., 2010). In these studies, walking speed is used as performance criterion indicating interference. Yet, even when walking speed was fixed by having subjects walk on a treadmill with constant velocity, interference by concurrent cognitive task could still be observed. Subjects reduced their step frequency at the cost of producing larger step lengths (Li et al., 2012). In situations with scarce processing capacity, prioritizing the motor task and allocating additional control resources to it, leads to reduced movement frequencies rather than a reduced movement amplitude.

Interestingly and in contrast, some researchers report an increase in movement frequency under dual-task conditions. In a study by van Impe et al. (2011), subjects drew circles within given space limits while concurrently performing a mental arithmetic task. Both, old and young subjects increased frequency of drawing movements under MCDT compared to ST conditions, which is interpreted as increased automatic control of the motor task when overall cognitive processing requirements are increased. Johannsen et al. (2013) had subjects perform ankle movements paced by a metronome while being engaged in *n*-back tasks of increasing difficulty. They observed an increase in movement frequency with increasing *n*-back difficulty. In addition, peak angular velocities were more regular with increasing velocity. However, with higher cognitive load, ankle movements were less strictly synchronized with the pacing stimulus (increased standard deviation of timing errors).

The increased movement frequency is called (involuntary) *hastening* by the authors and interpreted with “re-automatization.” When general cognitive processing capacities are occupied by an additional cognitive task, the motor task returns to a more or less automatic regime, relinquishing online control of synchrony with the pacing stimulus. By this, the system may drift toward a natural or preferred frequency. Hastening arises, when this eigenfrequency is faster than the pacemaker. This explanation of the hastening effect is based on three essential assumptions: (i) the existence of two different control regimes, an automatic and a cognitively controlled mode of operation, (ii) the idea that the processing of a cognitive secondary task interferes with this latter cognitive motor control processes, and (iii) the assumption that the automatic process controls the movement less strictly, that is, corrects errors less frequently and extensively.

Evidence for the existence of two motor control regimes for repetitive movements, an automatic and a cognitively controlled regime according to (i), where only the cognitive control mode shows interference under MCDT conditions according to (ii) comes from a study by Soylu and Newman (2016). They observed underadditive effects in brain activation in an fMRI study where subjects had to perform finger-tapping movements under ST and MCDT conditions (calculation). Based on this observation, they suspect that finger movements are controlled more automatically under MCDT conditions than under ST conditions. Holm et al. (2017) also studied tapping movements in continuation of a pacing signal. Increasing the load of a cognitive secondary task led to an increase in movement variability. However, this was only observed for movement frequencies higher than 1 Hz but not for lower frequencies. Interestingly, other authors also mention a threshold of 1 Hz as critical boundary between an automatic control of time intervals below 1 s and a rather cognitively controlled timing mode for above-second durations (Rammsayer and Troche, 2014). The assumption of two separate timing control mechanisms is also supported by physiological evidence, suggesting that automatic timing is controlled in motor areas, whereas cognitive control involves prefrontal areas (Lewis and Miall, 2003).

Furthermore, this differentiation also relates to another body of work, postulating different control structures (primitives), operating in different brain regions for rhythmic and discrete movements (Schaal et al., 2004). In this framework, discrete movements are defined as movements including postures at least before and after the movements and rhythmic movements are thought to be “recurrent movements with no stops” (Hogan and Sternad, 2007). In a study by Park et al. (2017), subjects were no longer able to perform oscillatory movements between two horizontal targets rhythmically and smoothly when the movement was gradually slowed down from 1 to 6 s per cycle.

Evidence in favor of assumption (iii) comes from work by Balasubramaniam et al. (2004). They find larger irregularities in trajectories of movements being performed in synchrony with a pacing signal compared to a condition without external pacing. These modulations of the movement patterns are likely to result from error compensation since the durations of movement phases show negative correlations. Importantly, this effect was modulated by movement frequency, the fastest movements showing the least amount of corrections. We have already mentioned that the amount of cognitive involvement in movement control might not just be modulated by movement duration but also depend on whether the movement can be considered as rhythmic or discrete. Indeed, Elliott et al. (2009) show stronger and faster corrections in discrete than in continuous (rhythmic) movements.

In the light the theoretical ideas and empirical observations mentioned so far, we may now have clearer expectations, under which conditions, the addition of a secondary cognitive task will severely impair performance in a motor task and in which settings interference should be smaller. One can even think of situations, where an additional cognitive task might even be beneficial. If motor performance is better when the automatic control regimes is left on its own and not disturbed by higher-order cognitive control, detracting the cognitive control away from the motor task by keeping it occupied with the cognitive task might be beneficial. A similar idea has already been propagated to explain the beneficial effect of an external attentional focus (Wulf, 2013) or the concept of ‘errorless learning’ (Maxwell et al., 2001; Poolton et al., 2005). The observed hastening effects, that is, the increased movement frequencies under MCDT conditions might be just one example of these boosting effects.

Yet, even though results reached significance, hastening effects were rather small. However, in most cases, the hastening effect was not the main focus of the experiment but rather a surprising side-effect, noticed by the researchers. Hence, the design was not always adequate to display the effect in the clearest possible way. In the studies by Balasubramaniam et al. (2004), van Impe et al. (2011), and Johannsen et al. (2013) the movement was externally paced. Therefore, any tendency to increase movement frequency is in conflict to the instruction to keep the beat. Remarkably, a tendency for hastening was still observed, even in spite of these strong diminishing factors.

In the present study, we wanted to see, whether the effect can be replicated under conditions designed to reduce diminishing factors and whether we can find support for the hypothetical explanation stated above. Due to this explanatory idea, hastening of repetitive movements should be strongest, when automatic control processes are involved. This should be more likely, for faster (period < 1 s) rhythmic movements, compared to slower (>1 s per cycle) discrete movements. In order to avoid the speed stabilizing effect of an external pacemaker, no desired frequency should be enforced externally. Instead, we selected tasks, where the task dynamics define a natural frequency range. We opted to use a paddleball task as a fast rhythmic task and a pegboard task as a relatively slow discrete repetitive task. We are well aware of the fact, that movement frequency is not the sole difference between these tasks. The pegboard task requires grasping and release of small objects, whereas the paddle is kept in hand throughout the whole movement, The spatial goal for the hand movements is defined by the layout of the pegboard, requiring significant spatial accuracy, whereas the paddleball task stresses temporal accuracy. Any of these differences might lead to specific effects on performance under dual-task conditions. Therefore, any result of our experiment cannot be understood as final proof of the “hasting-through-re-automatization” hypothesis. Nevertheless, this hypothesis allows us to derive very specific expectations in our experimental setting. Besides the pure behavioral effect of increased movement frequencies, we will also look at physiological evidence related to the underlying assumptions regarding automatization.

In order to control whether automatized processing is facilitated by detracting cognitive surveillance we also measured activity in the right prefrontal brain area. Other studies used fMRI-technology for this purpose. However, this strongly limits movements to rather restricted body positions and small movements of distal effectors (fingers or feed). In a systematic review, Leone et al. (2017) argued that NIRS technology is also suitable to validly quantify cortical activation changes during ST compared to MCDT. Since, NIRS measurements are also possible in our less restricted tasks we opted to use this method in our experiment.

Taken together, characterizing the pegboard task as a (relatively) slow discrete repetitive task and paddleball as fast rhythmic movement allows us to derive very specific expectations regarding behavioral effects (movement frequency) and brain activation changes under MCDT conditions. The following experiment was designed to test these predictions empirically.

## Materials and Methods

### Design

Given the theoretical background we have discussed so far, the frequency of a repetitive movement should be affected differently by a concurrent cognitive task depending on its frequency and its location along the discrete-rhythmic spectrum. In order to test this expectation, we selected two repetitive motor tasks, a paddleball task and a pegboard task, which should represent these categories. We measured subjects’ performance in these tasks and in the cognitive task (calculation) under ST and MCDT conditions. We also looked at brain activity in prefrontal areas using NIRS. Conditions were tested in a complete within-subject design in counterbalanced order across subjects and task conditions. For sake of completeness, we have to mention that we also tested other motor tasks in combination with the calculation task under MCDT conditions with the same subjects in the same experiment. However, these were not related to the current research question and we do not have any indication that subjects’ exposure to these additional conditions influenced the results reported here.

### Motor Tasks

*Paddleball* (Paddleball UNO, Active People, Binningen, Switzerland) is a one-handed rhythmical bouncing game. A small rubber ball is connected to the center of a hand-held paddle via an elastic band. The task is to bounce the ball so that it is repeatedly propelled off the paddle, then retracted back toward the paddle surface by the elastic band (17 cm) where it is hit again, thus starting the next cycle. Bouncing the ball in succession for as many repetitions as possible requires a rhythmic back and forth movement of the paddle adjusted to the movements of the ball. Note, although ball and hand movements have to be in synchrony, no external pacemaker is involved. The frequency of movement cycles evolves from the dynamical interaction of propelling and retracting forces. The system has no strict eigenfrequency since the amplitude of the paddling movement is variable. However, typical driving frequencies for the task are around (5 Hz). A bout of successive paddling cycles can be continued as long as the ball hits the paddle after being retracted. If the ball misses the paddle, an interruption occurs. Subjects have to restart a new bout by initiating a next first hit of the ball. This can be accomplished by different maneuvers, which we do not describe here. In fact, after the familiarization trials, each subject was capable to initiate a new bout very quickly, however using his/her own preferred technique.

**FIGURE 1:**
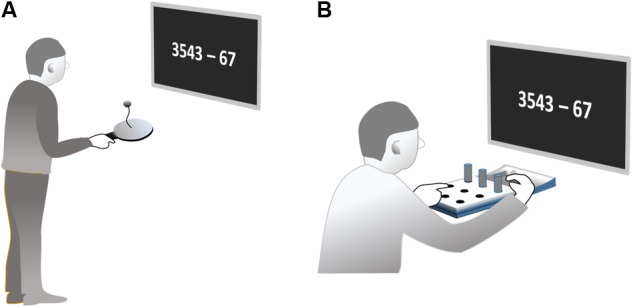
Posture and set-up of the paddleball task **(A)** and of the pegboard task **(B)**.

Subjects were asked to paddle with their right hand in an upright standing position for 60 s. They should try to stay in a paddling regime as long as possible. If a bout was interrupted, subjects were asked to start a new bout immediately. The task can be accomplished without necessarily visually fixating ball or paddle. Subjects were instructed not to control their movements visually, but rather look at a screen at eye level, positioned 50 cm in front of their eyes where the stimuli for the cognitive tasks were presented (**Figure 1A**). Movement frequency was measured acoustically based on the sound produced by each ball-paddle impact. Sound was recorded by an in-built computer microphone (8,000 Hz sampling rate, 8 bit per sample).

In the *pegboard task*, subjects sat at a table directly in front of a pegboard with nine holes arranged in a 3 × 3 square pattern (Jamar 9-hole peg test kit, Patterson Medical Ltd., Nottinghamshire, United Kingdom; **Figure 1B**). Nine cylindrical plastic pegs were placed in a hollow behind the board. Subjects were asked to use their right hand to put the nine pegs in the holes one by one. After completion, the pegs should be removed one by one until all pegs are in the hollow again. This filling and emptying of the holes should be continued without interruption throughout a period of 60 s. Note that movement frequency was also not externally paced in this task. The speed at which each single sub movement can be performed is at least partly curtailed by the distance traveled and the required precision at the endpoint according to Fitt’s Law.

In order to allow this task also to be performed without visual control, subjects were encouraged to use the left hand to detect the next empty hole exploiting tactile information. This freed their gaze to look at a screen in 50 cm distance from the eyes. Performance was measured by the number of stuck resp. drawn pegs, which was counted by a research assistant.

### Cognitive Task

The cognitive task was a mental subtraction task, presented on a screen. Subjects had to calculate the difference between a four-digit minuend and a two-digit subtrahend (e.g., 3543 – 67) as fast as possible and report the result verbally. Immediately after naming the result, a new digit pair was presented on the screen. Digit pairs were drawn from a list of selected 300 minuend-subtrahend pairs. The list did not contain digits equal to zero at the units and the tens position in neither minuend, subtrahend, or in the result in order to unitize difficulty. The digit pairs were presented in a single line in the format “3543 – 67” in the middle of the screen in white digits (∼3 cm height) on a black background. Subjects were asked to correctly solve as many subtractions as possible within the 60 s trial. Our raw measure of calculation performance was the number of correct subtractions in this interval (numCLC).

### Single and Dual-Task Conditions

The paddleball task and the pegboard task were executed in isolation (ST conditions, STpad and STpeg) and concurrently with the calculation task [dual-task conditions (DT), DTpad and DTpeg]. Under DT conditions, subjects were instructed to “perform both tasks as good as possible.” The calculation task was also performed without concurrent repetitive movement (STclc). In the STclc condition subjects remained in the posture required by the previous motor task, that is, if the STclc condition followed the paddleball task, subjects remained in an upright freehanded stance whereas subjects remained seated when the STclc condition followed the pegboard task. We analyzed STclc performance separately for the seated and the standing conditions. However, we found no indication of systematic differences between conditions.

### Participants

Fifteen students (nine females and six males; mean age = 27.1 years ± 7.1) participated in the study. All participants were right-handed according to their scores in the Edinburgh Handedness Inventory (Oldfield, 1971). The mean score was 88.7 ± 16.8. All subjects had normal or corrected to normal vision and self-reports indicated physical and mental health. All subjects gave written informed consent. Experimental procedures where in accordance with the declaration of Helsinki and were approved by the local ethics committee.

### Procedure

Overall, each participant completed three sessions on three consecutive days, a familiarization practice on the first day and three task blocks on each of the ensuing 2 days. Each task block was limited to one specific motor task. Hence, only one of these task blocks was dedicated to the pegboard task and one to the paddleball task. As mentioned above, four additional task blocks involving further motor tasks were executed. Since they were irrelevant for the current question, they will not be described in detail. We counterbalanced the order of the six task blocks across participants, also leading to a counterbalanced order of the pegboard and paddleball task.

Each task block contained nine trials: Every trial lasted 60 s and was followed by 90 s of rest, allowing physical and mental recreation. Blocks included three trials of the given motor task (STxxx), three repetitions of the calculation task (STclc), and three repetitions of the MCDT combination (DTxxx). The sequence of these nine trials within each task block was pseudorandomized to also counterbalance trial order within blocks across subjects and motor tasks.

To ensure that subjects were sufficiently familiar with the motor tasks when entering the task blocks on day 2, participants practiced each motor task three times for 60 s on day 1 under ST conditions. A reward (10, 20, 30 €) for the three best performers in the calculation task was announced to keep subjects’ motivation high throughout the entire experiment.

### Preprocessing of Behavioral Data

#### Paddling Performance

**FIGURE 2:**
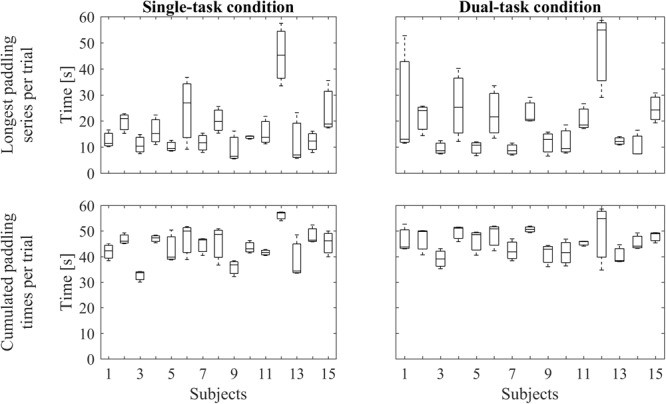
Paddling performance. The upper row represents boxplots of the longest individual paddling run per trial under single-task condition **(Left)** and under dual-task condition **(Right)**. The lower row depicts boxplots containing the cumulated durations of the individual paddling runs per trial under single-task condition **(Left)** and under dual-task condition **(Right)**. In every boxplot, data from three trials are included.

To record the series of paddleball hits we used the auditory signal and pre-processed it with Matlab 8.1 (MathWorks Inc., Natick, MA, United States). We filtered the signal with a fifth order, low pass Butterworth filter (cut off frequency: 10 Hz) to remove background noise and human voice. In the next step, all peaks indicating ball-paddle contacts were automatically detected, using a validated threshold. For analysis of paddling frequencies, we included bouts of at least five hits in a row, that is, it should last at least approximately 1 s. **Figure 2** shows individual paddling performances representing the longest paddling runs per trial and the summarized duration of all included paddling runs per trial, respectively. Paddleball frequency (frqPAD) was calculated by dividing the sum of all ball contacts across all (included) bouts by the respective summed duration of the (included) bouts. ΔfrqPAD is the difference between paddling frequencies between the STpad and the DTpad condition (ΔfrqPAD = frqPAD_DT_ -frqPAD_ST_).

#### Pegboard Performance

The frequency of movements in the pegboard task (frqPEG) was calculated as the number of moved pegs divided by task duration (60 s). The final dependent measure regarding our hastening hypothesis was the difference in frequency between the STpeg and the DTpeg condition (ΔfrqPEG = frqPEG_DT_ -frqPEG_ST_).

#### Calculation Performance

Taking the average number of correct answers per trial, under STclc condition subjects reached 4.2 ± 1.8 correct answers per minute. When concurrently playing paddleball, calculation performance changed to 3.6 ± 1.7 answers and it changed to 2.9 ± 1.5 correct responses during the pegboard task.

However, previous studies have shown that the number of correct subtractions in a 60 s trial (numCLC) substantially improves within 2 days of practice resulting in systematically higher performance with increasing trial number. This learning effect superimposes the effects of the actual task conditions and can be considered as systematic error variance in our case. We tried to eliminate this variance by fitting the calculation performance across trials within each subject with a logarithmic function (Newell et al., 2001). The residuals of this fit (resCLC) were then used to describe the effect of each test condition relative to the expected baseline performance after the respective amount of practice.

### Measurement of Cortical Load

We recorded neural activity in the prefrontal cortex of the right hemisphere with a one-channel (three light sources placed in a row), continuous wave (758 nm, 853 nm) fNIRS device (PortaLite, artinis, Elst, Netherlands). We fixed the sensor at Fp2 according to the international 10–20 EEG-system. The fNIRS systems emits infrared light of different wave lengths and measures the concentration of oxygenated and deoxygenated hemoglobin in the region of interest (ROI) based on the refraction profile. Based on the measured hemoglobin concentrations, the amount of brain-activity changes in the region can be estimated. Due to the limited number of channels, we cannot observe a broader range of cortical processes during MCDT across the entire brain. Nevertheless, our single-channel system allows us to monitor a specific brain area to observe its specific involvement in executive functions in different experimental conditions. In previous experiments, brain activity in prefrontal region monitored in our study showed a characteristic profile of increase under MCDT conditions that led us to believe that the region is involved in executive functions and task monitoring in the type of tasks we use in our experiment (Leone et al., 2017).

We recorded NIRS data with a sampling rate of 25 Hz over the entire test session, including all trials. In a first step, trials were visually inspected for movement artifacts before passing the data on for further analyses. Since fNIRS-signal recording in some trials of seven subjects seriously suffered from paddling while calculating due to facial expressions they were unable to avoid, the respective trials were eliminated from further analysis. In a second step, artifacts from different sources (heartbeat, eye-blinks, and evoked potentials) were removed using a Butterworth bandpass filter 0.05–0.8 Hz. In the next step, we averaged the refraction signals from the three sources for both chromophores oxygenated (HbO) and deoxygenated hemoglobin (HbR). We used NIRSTORM^1^ (Tadel et al., 2011) to perform the pre-processing of the fNIRS data. NIRSTORM is a publicly available plugin of the Matlab based software BRAINSTORM, used to analyze neurophysiological data.

It is not the absolute hemoglobin level but rather changes in hemoglobin concentration that are indicative of changes in brain activity due to the underlying neurovascular coupling. However, the change in concentration of the chromophores is only visible with a certain temporal delay. Therefore, we looked at the change in hemoglobin between trial start, that is, the average of the first 10 s (HbO_0-10_ resp. HbR_0-10_) within a trial and the average of the last 10 s of a trial (HbO_50-60_ resp. HbR_50-60_). We used these changes (ΔHbO = HbO_50-60_ - HbO_0-10_ and ΔHbR = HbR_50-60_ - HbR_0-10_) from start to end of a trial as measure of the cortical load induced by the respective condition (e.g., Mandrick et al., 2013b). Typically, an increase in HbO indicates an increase of cortical activity, whereas HbR should show a reciprocal decrease. Yet, this inverse relation of HbR is not always observed. Nevertheless, we follow the recommendations given by Obrig and Villringer (2003) and report the results of ΔHbR for sake of completeness, even though the hypotheses were less clear regarding this parameter.

### Statistical Analysis

We used one-tailed, one-sample *t*-tests to test the hypothesis whether movement frequency was increased under MCDT conditions for the paddleball task (ΔfrqPAD) and the pegboard task (ΔfrqPEG) separately. A one-factorial ANOVA with repeated measurements of the dependent variable “cognitive performance” (resCLC) across levels of the independent variable “task” (levels: STclc/DTpeg/DTpad) was computed to check to which extent cognitive performance is affected by the type of concurrent motor activity.

Whether changes in brain activity (ΔHbO, resp. ΔHbR) changed differently across conditions (independent variable “condition” with levels: STclc/STxxx/DTxxx), for the two motor tasks included in this study (independent variable “task,” levels: Pegboard/Paddleball) was tested by a two-factorial ANOVA with repeated measurement on both factors. As already mentioned, our focus was mainly on the dependent variable ΔHbO. However, we will also report the equivalent results for ΔHbR. *Post hoc* analyses checked for differences using Bonferroni corrected *t*-tests. Significance level was set to *p* = 0.05 in all inferential statistical tests.

Further, we were interested in whether cognitive and/or motor performance is systematically related to cortical load. We quantified the strength of this connection by calculating correlations between the motor performance (frqPAD_ST_, frqPAD_DT_, frqPEG_ST_, and frqPAD_DT_) resp. cognitive performance (numCLC_ST_, numCLC_DTpad_, and numCLC_DTpeg_) and the changes in prefrontal activation (ΔHbO) in a trial-wise manner. Each correlation reported in **Table 1** is based on *n* times three data pairs, resulting from *n* subjects with three trials per subject.

**Table 1:** Correlations between cortical load and measures of cognitive and motor performance.

	Paddleball task block			Pegboard task block		
Condition	*n*	Motor task	Cognitive task	*n*	Motor task	Cognitive task
Single task	15	–0.11	–0.02	15	–0.50^∗∗∗^	–0.12
Dual task	8	0.12	–0.17	15	–0.40^∗∗^	–0.38^∗∗^

*Cells differ in sample size (*n*) since seven subjects had to be excluded from analysis due to movement related artifacts in the fNIRS signal in the paddling dual-task condition. ^∗∗^*p* < 0.01, ^∗∗∗^*p* < 0.001.*

## Results

### Motor Performance

**FIGURE 3:**
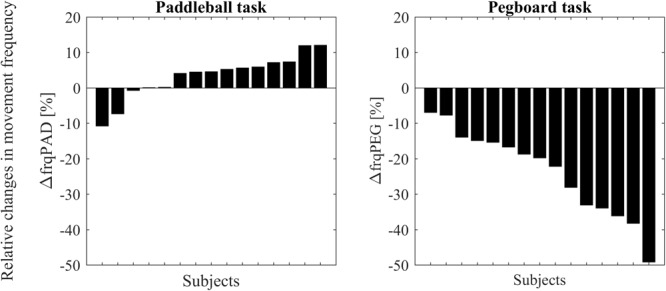
Changes in movement frequencies from single to dual-task condition for all subjects. Subjects are ordered according to effect size. Results for the paddleball task (ΔfrqPAD expressed as % of individual mean frequency) are displayed on the left side. Results (ΔfrqPEG expressed as % of individual mean frequency) for the pegboard task on the right side.

A *t*-test showed that movement frequency was significantly reduced under DT conditions compared to ST in the pegboard task [*t*(14) = -7.289, *p* < 0.001, **Figure 3**]. In contrast, as hypothesized, the paddleball task shows hastening, that is an increased movement frequency (on average 3% faster) under DT conditions [one-tailed analysis: *t*(14) = 1.7662, *p* = 0.0496]. Only two of our 15 subjects had substantially slower frequencies under DT conditions, whereas ten showed clearly increased frequencies. These were up to approximately 10% higher.

### Cognitive Performance

**FIGURE 4:**
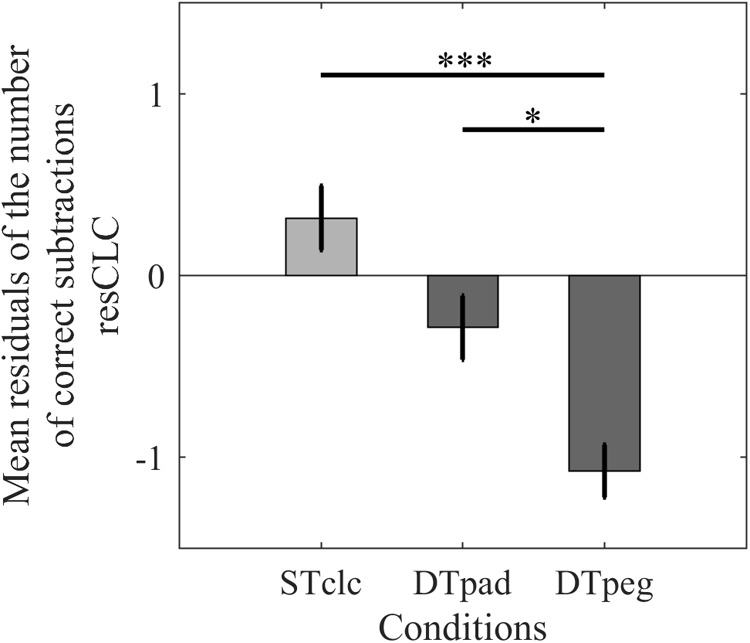
Cognitive performance as mean relative number of correct calculations (resCLC) for single-task calculation (STclc) and for the two dual-task conditions (Paddleball: DTpad; Pegboard: DTpeg). Error bars represent standard errors of the mean. ^∗^*p* < 0.05, ^∗∗∗^*p* < 0.001.

Cognitive performance (resCLC) differed significantly across tasks [*F*(2,28) = 11.293, *p* < 0.001, η^2^= 0.446; **Figure 4**]. *Post hoc* analyses indicated that performance suffered when the calculation task was performed simultaneously with the pegboard task compared to STclc (*p* < 0.001), whereas cognitive performance was not as strongly reduced when subjects paddled while calculating (*p* = 0.23). *Post hoc* test even revealed that cognitive performance is better while paddling compared to the pegboard condition (one-tailed analysis: *p* = 0.0475). According to these results, with respect to cognitive performance, the paddling condition is more similar to the STclc condition than to the pegboard condition.

### Cortical Load

**FIGURE 5:**
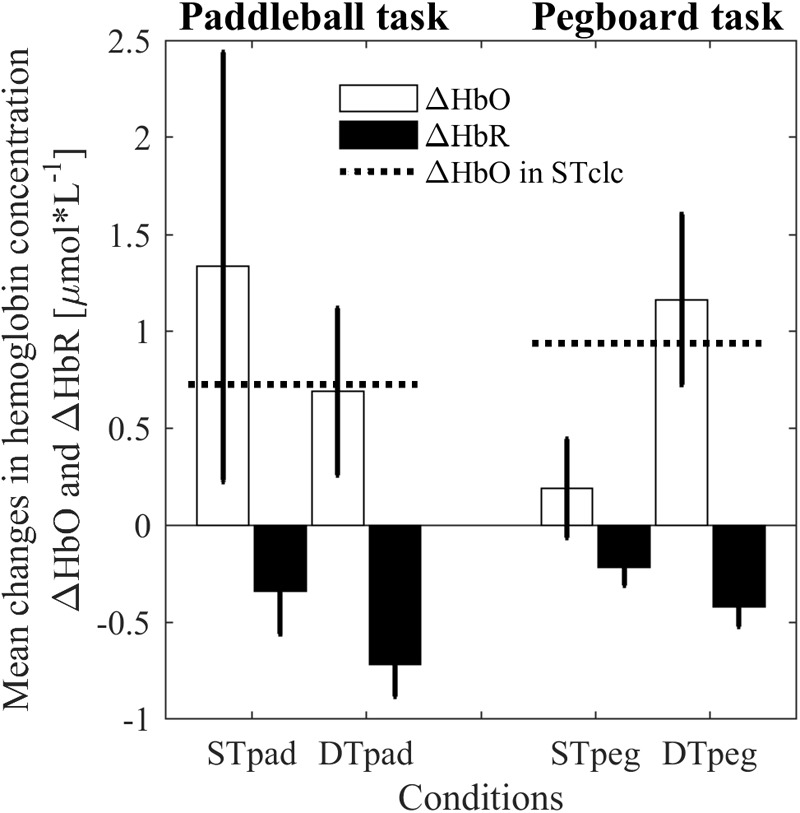
Changes in hemoglobin concentrations for HbO (white bars) and HbR (black bars) as indication of processing load in prefrontal brain region for paddleball (pad) and pegboard (peg) task under single (ST) und dual-task (DT) conditions. The dashed horizontal lines represent cortical activity induced by single-task calculations (STclc). Error bars represent standard errors of the mean.

The ANOVA revealed no main effects for the dependent variable ΔHbO. There is neither a “condition” effect [*F*(2,14) = 0.209, *p* = 0.814, η^2^ = 0.029] nor a “task” effect [*F*(1,7) = 3.67, *p* = 0.564, η^2^ = 0.050]. However, as expected from our hypothesis, we found a significant interaction [*F*(2,14) = 3.814, *p* = 0.048, η^2^ = 0.353]. Whereas cortical load is higher for the paddleball task under ST conditions (ΔHbO_STpad_> ΔHbO_STpeg_), the relation is reversed under DT conditions (ΔHbO_DTpad_ < ΔHbO_DTpeg_; **Figure 5**). As a by-product, we also clearly see, that both STclc conditions show similar activation changes [*t*(14) = 0.834; *p* = 0.4183]. This observation indicates that cortical load does not depend on the posture taken while calculating (standing vs sitting). Interestingly, the cortical load in the DTpad condition is almost identical to the ST calculation condition (STclc; **Figure 5**). Apparently, paddling does not add any further cortical load to calculation.

In HbR we found a significant “task” effect [*F*(1,7) = 8.799, *p* = 0.021, η^2^ = 0.557], but no effect of “condition” [*F*(1.246,8.722) = 0.542, *p* = 0.519, η^2^ = 0.072] and no significant interaction effect [*F*(2,14) = 1.504, *p* = 0.256, η^2^ = 0.177]. HbR was decreased more strongly in the paddleball task blocks compared to the pegboard task block. However, as mentioned before, we did not have clear *a priori* hypotheses regarding the behavior of HbR.

### Correlation Analyses

**Table 1** shows correlation between the measures of cortical load (ΔHbO_STpad_, ΔHbO_DTpad_, ΔHbO_STpeg_, ΔHbO_DTpeg_, and ΔHbO_STclc_,) and measures of cognitive performance (numCLC_ST_, numCLC_DTpad_, and numCLC_DTpeg_) resp. motor performance (frqPAD_ST_, frqPAD_DT_, frqPEG_ST_, and frqPAD_DT_). A negative correlation indicates that higher cortical loads are associated with lower performance. A previous study already showed that such a negative correlation is observed when the system operates at its limits, especially under DT conditions (Mirelman et al., 2014). The more performance falls back against an internal reference, the more prefrontal activity is increased during the 60 s interval. This is particularly the case for the pegboard task, relatively low motor performances are systematically linked to higher increases in activation in ST and in the DT condition. For the cognitive task, this connection can only be seen under DT conditions. Notably, for the paddleball task, neither of these relations are observed. We will discuss the relevance of these observations later in more detail.

## Discussion

The main goal of our experiments was to test specific predictions derived from the “hasting-through-re-automatization” hypothesis because of a particular classification of our experimental tasks. According to the literature, we expected a hastening effect, that is, a frequency increase under MCDT conditions only for one of our tasks, but not for the other, both belonging to different specific sub-classes of repetitive movements. According to the explanatory concept outlined so far, fast (>1 Hz) repetitive rhythmic movements should be controlled by an automatic control regime, which, however, is supervised by higher cognitive control processes. The involvement of the cognitive processes may lead to interference and probably also to a slower movement execution. Though, when a concurrent cognitive task binds all cognitive resources, the automatic process is freed from the detrimental effect of cognitive surveillance, allowing higher movement frequencies in that situation.

The results of our experiment were well in line with the expectations derived from this concept. We indeed observed a significant increase in movement frequency for the fast rhythmic movement (paddleball) under MCDT conditions in our experiment, thus replicating the findings reported by van Impe et al. (2011). In contrast, there was no such effect for the slow discrete repetitive movement (pegboard) in our experiment. Movement frequencies were clearly reduced under MCDT conditions in the pegboard task. The basic assumption underlying the explanation of this phenomenon is that both tasks are controlled by different types of control regime. This is confirmed by the observation that cortical load increases from STpeg to DTpeg but not from STpad to DTpad. In the latter case, one can even see a drop in prefrontal brain activation to the level of the cognitive ST. Actually this is exactly what you would expect, if the motor task runs automatically, that is, without any additional, prefrontally located cognitive processes being involved. By this, cortical activity is not upregulated to its limits in the paddleball task, even under MCDT conditions. Quite the contrary, in the pegboard task, adding the calculation task further increased cortical activity. As the correlational results show, this upregulation reaches a level where signs of saturation become visible. Surprisingly however, we also saw negative correlations between increase in prefrontal activity and motor performance in the ST–pegboard condition, where the absolute activation level was far from maximum. We do not know yet, how to explain this particular finding.

Even though the outcomes of our experiment are mostly well in line with our expectations, we need to mention some limitations of the current study. Most of the studies reporting hastening effects relied on an external pacemaker, potentially attenuating the hastening effect due to its normative function. In our study, we tried to overcome this limitation by using movements in which frequency is not externally set but rather the result of the internal and external task dynamics.

Another limitation of previous studies arises from the fact that they were mostly done in a brain scanner, allowing only very restricted movements. We wanted to overcome this limitation by studying rather naturalistic movements with larger movement extents. Consequently, we used fNIRS to still be able to collect physiological correlates of brain activity. Even though this was mostly successful, we have to admit that we encountered serious movement artifacts in some of our subjects when they paddled vigorously, particularly in MCDT condition. Only for about half of our subjects, the NIRS data were sufficiently clean, to include them in our analyses. This strongly reduced the power of our study. The other half showed strong head movement resp. strong mimic activity, partly due to the experienced satisfaction resp. dissatisfaction with subjects’ own performance. This should be better controlled in future studies.

The recording of paddling movements based on the acoustic events was successful in registering ball impacts on the paddle. However, we were unable to analyze the recovery movements, that is, the movements used to initiate a new paddling bout, once the previous one could not sufficiently be continued.

Despite these methodological limitations, the overall result profile strongly supports the idea that a specific type of repetitive movements might indeed benefit when its control is freed from cognitive surveillance, which might be accomplished by another concurrently performed cognitive task. This is possible when an automatic control regime is available to master the task even in absence of higher cognitive control. However, when the repetitive task itself requires substantial cognitive control, an additional cognitive task will have detrimental effects. In our study, we have only looked at one representative of these movement classes and received a result profile that is well in line with the explanatory concept. Yet, alternative explanations based on further differences between the two tasks studied in our experiment cannot definitely be ruled out. Therefore, further empirical evidence is required to substantiate the “hasting-through-re-automatization” hypothesis.

## Author Contributions

CL and HM gave substantial contributions to the conception and design of the work. They approved the final version to be published. For all aspects of the work in ensuring that questions related to the accuracy or integrity of any part of the work are appropriately investigated and resolved by them and declare to be accountable. Interpretation of data and drafting of the manuscript was done conjointly by them, both bringing in important intellectual content. CL was mainly responsible for data collection and analysis.

## Conflict of Interest Statement

The authors declare that the research was conducted in the absence of any commercial or financial relationships that could be construed as a potential conflict of interest.

## Footnotes

1 http://github.com/nirstorm
